# Supplementary material for: Interleaving asynchronous and synchronous activity in balanced cortical networks with short term synaptic depression
Source: Nat Commun. 2025 Sep 30;16:8657. doi: 10.1038/s41467-025-63818-z (PMC12484685; doi:10.1038/s41467-025-63818-z)
Supplement: Supplementary file 1 — Supplementary Information [file 41467_2025_63818_MOESM1_ESM.pdf]

Supplementary Materials

Finite Size Scaling of Rare Population Events

The statistics of population events from our model behaves as expected for rare processes. In the $K \to \infty$ limit population-wide fluctuations vanish and consequently population events will never occur. As K decreases, fluctuations increase and thus events become more and more likely, until finally for low enough K the finite size network has behavior significantly different from the large K limit (Fig. S1a). A hallmark of rare noise induced transitions is an exponentially distributed inter-event interval[59]. Over a large range of K our model agrees (Fig. S1b), and the exponential parameter grows exponentially with system size (Fig. S1b inset), again in agreement with rare event theory due to finite system size[59,60,61,20,62,63]. Satisfyingly, exponentially distributed inter-event intervals are found in our *in vitro* data, aggregated over all events and all slices (Fig. S1c). Moreover, qualitative agreement in inter-event interval distribution is found between the model and the data for reasonably sized networks (K on the order of a few hundred). This is in contrast to the traditional $1/K$ scaling, where rare events are often observed only for exceptionally small networks, with K on the order of tens. In total, the circuit model in Eqs. (1)–(3) recapitulates the main features of the asynchronous and population event epochs of the spontaneously active cortical slice experiments.

Suite of Population Event Dynamics in the Spiking Neuron Model

The rate-based model exhibits six distinct dynamical behaviors based on synaptic properties: Low state, Excitable regime, Bistable regime, Periodic dynamics, Oscillatory population events, and a Saturated state (see Fig. 7). Our analysis determines how threshold parameters $\{\theta_{EE}, \theta_{IE}\}$ govern these dynamics, identifying bifurcations that separate these distinct behaviors. However, the spiking neuron model, due to the complexity of the parameters controlling synaptic properties, makes formal calculations of these bifurcation boundaries challenging. However, by carefully adjusting these parameters within our theoretical framework, we have successfully replicated each predicted dynamic (see Fig. S2).

In the main text we showed that the network of spiking neuron models has a Low state (Fig. 10d), an Excitable state (Fig. 10c), and can produce Oscillatory population events (Fig. 10c). Here, we focus on demonstrating the remaining three dynamics in the network of spiking neuron models: Periodic dynamics (Fig. S2b), a Saturated state (Fig. S2c), and a Bistable state (Fig. S2d).

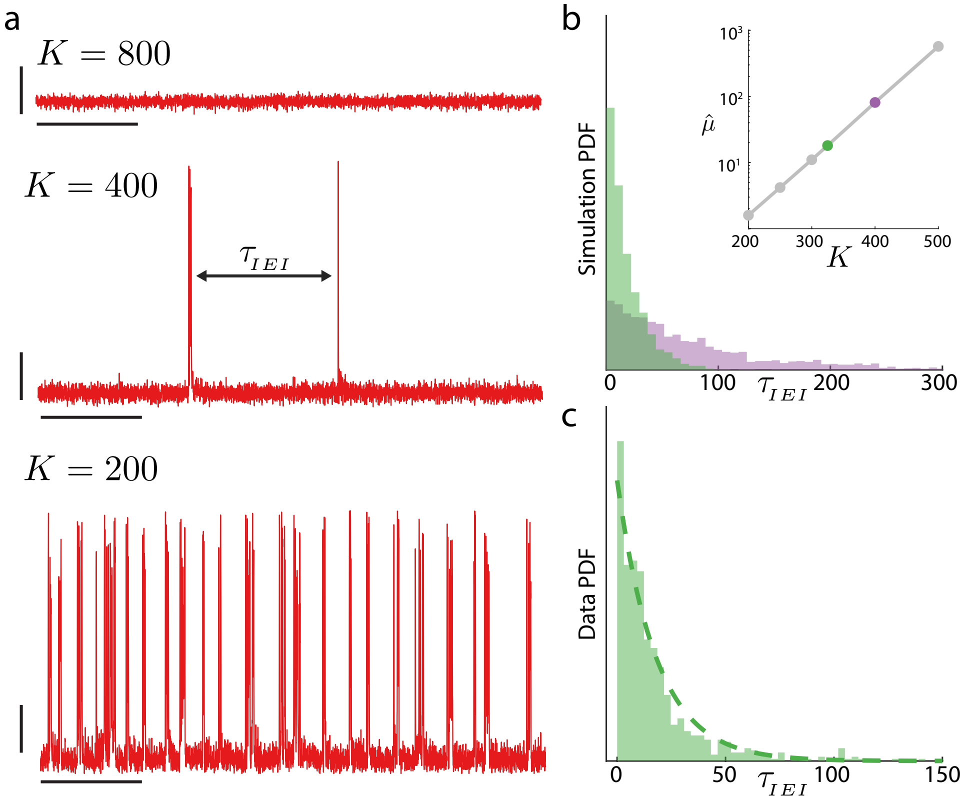

Figure S1: Population level fluctuations due to finite network size induces population events. **a:** Sample simulation trajectories of the excitatory population activity for three different system sizes K. Vertical scale bars indicate 0.1 a.u., and horizontal scale bars indicate 10s. Each trace shows a total of 50s of simulation. As K increases, events become exponentially less likely. τ_{IEI} is the inter-event-interval measuring the time between two successive events. **b:** τ_{IEI} is exponentially distributed, consistent with a noise-induced escape model. $\hat{\mu}$ is the exponential parameter fit from a maximum likelihood estimate (MLE) of simulated data; as expected $\hat{\mu} \sim \exp(K)$. Highlighted are distributions for $K = 325$ (green) and $K = 400$ (purple). Connection strengths between populations were scaled for these simulations to fix the underlying vector field to isolate the system-size/noise relationship. Histograms are scaled to have total area 1. **c:** Distribution of inter-event times from recorded *in vitro* data (Figs. 1 and 2). The distribution appears exponentially distributed, despite being aggregated from all slices. Dashed line is the MLE fit to the data, giving approximately the same $\hat{\mu}$ as the simulation for $K = 325$ (Data: $\hat{\mu} = 18.03$, Sim: $\hat{\mu} = 18.05$).

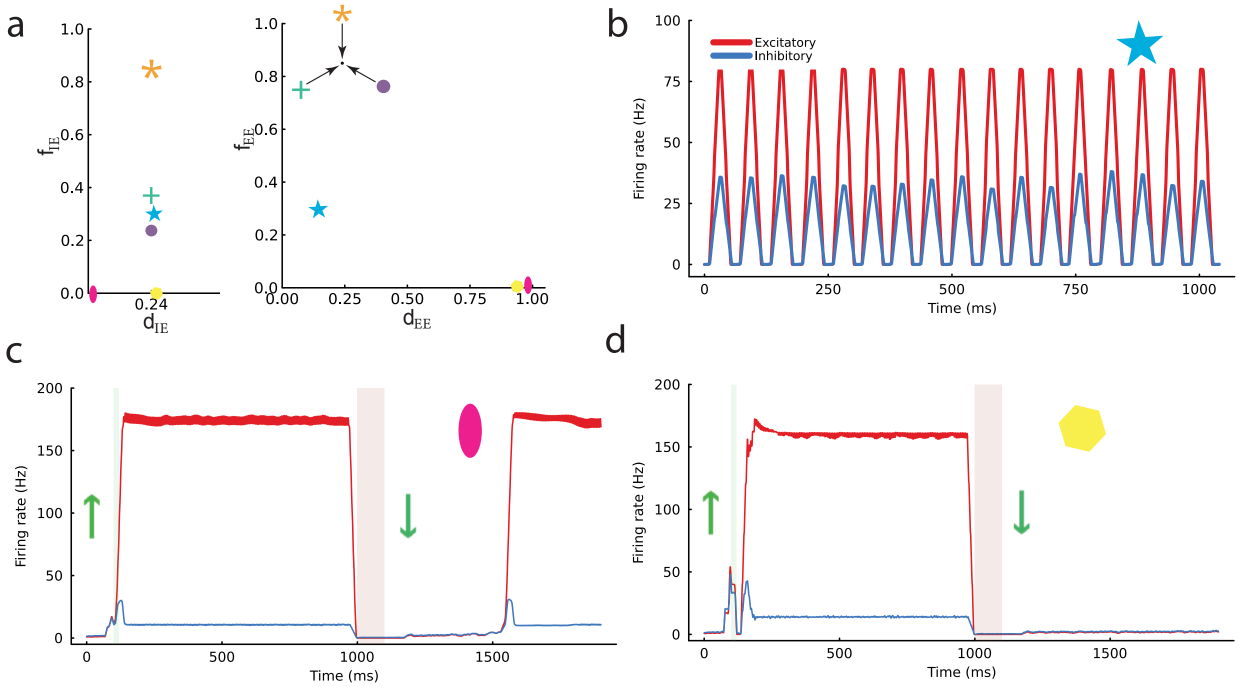

Figure S2: Suite of network dynamics possible in the spiking neuron model. **a:** Plasticity parameters of the $E \to I$ and $E \to E$ synapses for each scenario. Symbols in the top right corner of each plot represent the parameter settings used in simulations: (+) for Excitable, (o) for Oscillatory event, (*) for Low state, as previously shown in Fig. 10. Here, (pentagram) denotes Periodic dynamics, (ellipse) denotes a Bistable regime, and (polygon) denotes a Saturated state. **b:** Example simulations demonstrating Periodic dynamics (without external stimuli). **c:** Simulations showing a Saturated state. Initially, a positive external stimulation is applied to the excitatory population for 20 ms, facilitating the network's transition to a high firing rate. Subsequent negative stimulation fails to alter the network's state, illustrating its stability. **d:** Demonstration of the Bistable regime. Following a positive stimulation, the network reaches a high firing rate stable fixed point. A later negative stimulation leads the network to settle into a low firing rate stable fixed point.

Simulation Parameters

Parameter	Value	Description
N_E	4000	Number of excitatory neurons
N_I	1000	Number of inhibitory neurons
V_{th}	1 (scaled)	Threshold potential
V_{re}	0 (scaled)	Reset potential
τ_{ref}	5 ms	Refractory period
μ_E^L, μ_E^H	1.1, 1.2 (scaled)	Range for excitatory resting potential
μ_I^L, μ_I^H	1.0, 1.05 (scaled)	Range for inhibitory resting potential
τ_E	15 ms	Excitatory membrane time constant
τ_I	10 ms	Inhibitory membrane time constant
σ_E, σ_I	0.3	Noise intensity
τ_E^r, τ_E^d	1 ms, 3 ms	Excitatory synaptic rise and decay time constants
τ_I^r, τ_I^d	1 ms, 2 ms	Inhibitory synaptic rise and decay time constants
$\bar{J}_{IE}$	0.014	Baseline synaptic strength ($E \to I$)
$\bar{J}_{EI}$	-0.051	Baseline synaptic strength ($I \to E$)
$\bar{J}_{II}$	-0.057	Baseline synaptic strength ($I \to I$)
$\bar{J}_{EE}$	0.024	Baseline synaptic strength ($E \to E$)
τ^D	103 ms	Depression factor decay time constant
τ^F	96 ms	Facilitation factor decay time constant
χ_{EE}	0.2	$E \to E$ connection probability
$\chi_{EI}, \chi_{IE}, \chi_{II}$	0.5	Probabilities of connection for $I \to E$, $E \to I$, and $I \to I$ neurons
T	2500 ms	length of simulations

Supplementary Table 1: Network model parameters and their values.
